# Decomposition stages as a clue for estimating the post-mortem interval in carcasses and providing accurate bird collision rates

**DOI:** 10.1038/s41598-022-20628-3

**Published:** 2022-09-28

**Authors:** Virginia Moraleda, Julia Gómez-Catasús, Claudia Schuster, Luis M. Carrascal

**Affiliations:** 1 Grupo de Rehabilitación de la Fauna Autóctona y su Hábitat, GREFA, Majadahonda, Madrid Spain; 2 Novia University of Applied Sciences, Raseborgvägen 9, 10600 Ekenäs, Finland; 3 Terrestrial Ecology Group, Department of Ecology, Universidad Autónoma de Madrid (TEG-UAM), 28049 Madrid, Spain; 4 Centro de Investigación en Biodiversidad y Cambio Global, Universidad Autónoma de Madrid (CIBC-UAM), 28049 Madrid, Spain; 5 Departamento de Ecología Evolutiva, Museo Nacional de Ciencias Naturales (MNCN-CSIC), 28006 Madrid, Spain

**Keywords:** Ecology, Environmental sciences

## Abstract

The estimation of the post-mortem interval is crucial to accurately provide bird collision rates against manmade infrastructures. Standard methodologies recommend initially clearing all carcasses to ensure that subsequent collisions can be attributed to known time intervals. In this study, we propose a more cost-efficient approach aiming to link the decomposition stages as unequivocally as possible to the most likely time elapsed since death. Factors influencing the decomposition stages of bird carcasses were evaluated by means of two experiments. Firstly, we examined carcasses of large birds in three seasons differing in temperature, sun radiation and humidity: summer, autumn and spring. Secondly, we tested the influence of body mass in the same season (spring) using small, medium-sized and large bird carcasses. Results showed that the decomposition score increased monotonically with time, attaining the highest magnitude effect. A carcass with a decomposition score ≥ 4 (skeletal reduction) was in the field for ≥ 15 days, whereas a carcass with a score < 3 (fresh or emphysematous) was exposed < 3 days. Decomposition scores were higher in summer and did not differ among carcass sizes. This study provides an alternative protocol to estimate the post-mortem interval in wild birds in studies in search of bird fatalities.

## Introduction

Estimates of avian collision rates with power lines and other human infrastructures are commonly based on carcass counts^1,2^. The estimation of the post-mortem interval (e.g., time that has passed since death after collision) is particularly important to accurately provide the number of fatalities per unit of time (e.g., carcasses per year or month^3^). Accordingly, standard methodologies recommend initially clearing all carcasses under the power lines (see for example^4–7^), to ensure that subsequent collisions could be attributed to known time intervals. This methodology, though ideal and advisable, has several limitations: (1) it is highly-demanding from a financial and logistic point of view, or unapproachable when hundreds of kilometres have to be covered considering the long time elapsed since the beginning of the removal of the carcasses and the beginning of carcass counts; (2) if the presence of environmental agents is mandatory to remove the carcasses of protected species (as is the case of Spanish legislation), researchers are subjected to the time schedules and availability of authorised environmental officers in the removal of carcasses, not necessarily compatible with the sampling schemes and field-work; and (3) the time invested in previous carcass removal does not ensure that all carcasses are found and removed given the demonstrated biases in carcass detection, and the low probability of detection of small animals^1,8,9^. When the prior removal of carcasses poses logistical and economic difficulties or is not possible, it is necessary to have shortcuts that allow dating the time elapsed since the fatalities, thus helping to discriminate between recent or older carcasses, and avoiding a paramount investment of time and economic resources prior to the final sampling of carcasses. The development of these efficient and innovative approaches to accurately estimate bird fatalities based on carcass searches, with their related correction factors, has been proposed as a priority area for research for the study and quantification of bird collisions with power lines^10^.

Immediately after death a sequence of post-mortem changes, both physical and chemical, occur in the dead body leading to different decomposition stages^11,12^. These changes occur gradually and sequentially as the time since death increases^12^. Therefore, gaining insights into the decomposition stages of carcasses is crucial for the estimation of post-mortem intervals, which in fact affect carcass persistence and the assessment of infrastructure-driven mortality, thus masking its impact on wildlife^1,13^. Furthermore, a myriad of intrinsic and extrinsic factors may also influence the process of decomposition, altering the onset and duration of each decomposition stage^14^. Intrinsic factors include carcass size, body fat, presence of wounds, bruises or fractures, among others^15–17^; whereas extrinsic factors encompass environmental aspects such as temperature, moisture, oxygen tension, or insect and scavenger activity^14,18,19^. Decomposition stages, and the factors influencing them, have been extensively addressed in mammals due to their relevance in human death investigations (see for example Pittner et al.^19^ and Probst et al.^20^); however, little information is available for birds^17,21,22^. Measuring how bird carcass decomposition evolves over time, as well as the influencing factors, might provide accurate estimations of the time since death, and ultimately reliable estimates of bird fatality rates within controlled time spans.

In this paper, we propose a more cost-efficient approach to the monitoring of mortality estimates in power lines that allows for the accurate estimation of the time since fatalities. Although our study focuses on bird collision fatalities at power lines, its conclusions also have application for other monitoring schemes of infrastructure-driven mortality, such as roadkill or windfarm surveys. The main goal of this paper is to link the decomposition stages as unequivocally as possible to the most likely time elapsed since death (e.g., collision fatality). To this end, we evaluated several factors influencing the decomposition stages of bird carcasses by means of two carcass decomposition experiments carried out on the two easternmost islands of the Canary archipelago, Fuerteventura and Lanzarote. We tested for the effects of time elapsed since carcass placement, carcass size, season according to changes in temperature, solar radiation and rainfall, and large spatial variation on decomposition scores related to the decomposition process^14,19^. We predicted that the time elapsed since carcass placement would be highly approachable by the decomposition stages^11,18^. We also expected that changes in the decomposition scores would be accelerated in small-sized carcasses as compared to large carcasses^16,23^. The decay rate would be hastened in seasons defined by higher: (1) temperature^24^ and humidity^15,25^ due to their positive effect on insect activity^26–28^; and (2) sun radiation^20^ due to its influence on the breakdown of tissues that facilitate the posterior microbial activity (see also Gliksman et al.^29^ and Araujo et al.^30^ for litter decomposition in drylands). Finally, we also analysed the contribution of spatial variation among carcass locations, assessing the contribution of differences between sites within each island, and between the two islands to the observed variation in the decomposition rates.

## Material and methods

### Study area

This study was carried out on the two easternmost islands of the Canarian archipelago: Lanzarote (846 km^2^, 670 m a.s.l.; 29° 2′ 6″ N, 13° 37′ 58.8″ W) and Fuerteventura (1660 km^2^, 807 m a.s.l.; 28° 25′ 27″ N, 14° 0′ 11″ W). The climate is semi-arid with mean temperatures ranging from 14 to 29 °C and annual precipitation below 200 mm. These landscapes are dominated by scarcely vegetated arid and semi-arid habitats, as the result of aridity, lithology (volcanic soils), grazing by goats (mainly Fuerteventura), human uses (e.g., cultivated fields), terrain aspect and slope. The plant community is dominated by xerophytic shrubs such as *Launaea arborescens*, *Lycium intricatum*, *Salsola vermiculata*, *Suaeda spp*. and *Euphorbia spp*. For a detailed explanation of the study area see Fernández-Palacios and Martín-Esquivel^31^ and Gómez-Catasús et al.^8^.

### Decomposition rate experiment

We carried out two experiments to assess the decomposition process in bird carcasses. Carcasses were placed in six sites in Lanzarote and nine sites in Fuerteventura. The sites were spread throughout the two islands in order to embrace the whole environmental variability, since several factors are expected to affect the decomposition process^14,19^. The minimum distance between the two nearest sites in Lanzarote was 3.1 km, while it was 6.0 km in Fuerteventura. The carcasses were chicks of domestic hens *Gallus gallus domesticus* (small), Rock Pigeon *Columba livia* or Common quail *Coturnix coturnix* (medium) and adult hens (large). Carcasses were kindly supplied by Oasis Park zoo (Fuerteventura). They were frozen immediately after death in order to avoid different decomposition rates, and they were defrosted at ambient temperature the day before placement (category 0). The decomposition-degradation state of carcasses after the placement in the filed were established beforehand in five categories: (1) *fresh*, characterised by the presence of soft tissues before the initial body inflammation due to bacterial action; (2) *emphysematous*, from showing very apparent inflammation to skin rupture due to internal gas pressure and superficial tissue decomposition; (3) *colicuative*, encompassed advanced decomposition and disappearance of soft tissues; (4) *post-colicuative*, only dried tissues, cartilages and bones were present; and (5) *skeletal reduction*, referred to the mere occurrence of bone and feather remains (Table 1; Appendix A).

**Table 1:** Decomposition–degradation states.

Decomposition–degradation state	Physical description	Cadaveric fauna
Fresh	Presence of soft tissues, from death to body inflammation due to bacterial fermentation. Indicators of the freshness of the carcass are the presence of fresh blood or the good condition of feathers that do not easily detach from the body	Flies and wasps were the initial colonizers of the carcasses, but also beetles, ants and eggs deposited in oral cavities, eyes, open wounds, or cloaca. First instar larvae can also be found
Emphysematous	From the beginning of inflammation caused by bacterial fermentation until body rupture due to pressure and superficial tissue decomposition. Carcasses are found swollen, and in some cases fresh blood is still present	Ants, beetles, and flies in adult stages, but also in egg stages. Increase of the proportion of larvae which are found at different stages and therefore of different sizes
Colicuative	Encompass from gas outlet until decomposition and disappearance of soft tissues. Little skin remains, and the bones and tendons are still moist. The feathers are in bad conditions or totally degraded in some parts	Higher proportion of beetles than in previous stages, and to a much lesser extent flies and ants. This is the most active phase of the larvae of necrophagous insects and thus, many larvae can be found in different stages (and sizes) and pupating individuals appear for the first time
Post-colicuative	The carcasses are hollow and mummified, with dried tissues, cartilages, and bones and some of the bigger feathers still present	Individuals in pupal stages and, to a lesser extent, in larvae stages. On some occasions, individuals of Diptera starting a new life cycle (adults, eggs and larvae) can be found
Skeletal reduction	Only bone remains are distinguishable, sometimes a few wing feathers	The cadaveric fauna significantly decreases at this stage. Flies disappear. Mainly beetles. Individuals in larval or pupal stages are rarely found. Mites and arachnids appear for the first time

For each state the physical changes are indicated, as well as the associated cadaveric fauna. See Appendix A for further information and photographs.

In the first experiment regarding large carcasses, we placed two bird carcasses per site separated by at least 20 m, in three different seasons considering precipitation and ambient temperature: summer (July 2015; dry and hot with high levels of solar radiation; 30 carcasses), late autumn (November–December 2015; wet and cool, with less sun radiation due to cloudiness and lower height of the sun above the horizon; 30 carcasses) and early spring (March 2016; with intermediate environmental conditions; 30 carcasses). We georeferenced the location of each carcass with a portable GPS for subsequent visits. Carcasses were monitored on five occasions: 1, 3, 7, 15 and 30 days after placement in the field. We recorded the decomposition-degradation score of each carcass on each visit, assigning on some occasions half scores for intermediate states, especially for *colicuative*/*post-colicuative* and *post-colicuative/skeletal reduction* states. All remains were removed on the day 30 of the experimental trials. Eighteen out of 30 carcasses remained in the field up to day 30 in summer, 25 in late autumn and 25 in early spring.

In the second experiment carried out in early spring, we added two or three carcasses (according to carcass availability) of small and medium-sized birds in each sampling site where large carcasses of hens were located (separated by at least 20 m and totalling 90 carcasses). Due to the high rates of disappearance of small and medium-sized carcasses (see Gómez-Catasús et al.^8^), the temporal variation of the decomposition-degradation state could not be evaluated until day 30 due to small sample sizes, and thus low statistical power. Only one out of 30 carcasses of small-sized birds, and three out 30 of medium-sized birds, remained in the field up to day 30. This is why the second experiment, dealing with the effect of body size on the decomposition-degradation score, was restricted to days 1, 3 and 7.

### Statistical analyses

Data on decomposition scores were analyzed by means of general mixed effects models, using random intercept and random slopes models. Sampling sites were treated as a categorical random factor with 15 levels (six sites in Lanzarote and nine in Fuerteventura), thus defining the true, non-pseudoreplicative, sample unit. The model analyzing the first experiment only with large-sized carcasses included the island (two levels), season (three levels) and the days since placement of the carcasses in the field (five ordered levels, from day 1 to day 30), and their corresponding three two-way interactions, and one three-way interaction. The model analyzing the second experiment with the three bird sizes in early spring included the island (two levels), body size (three levels) and the days since placement of the carcasses in the field (three ordered levels, from day 1 to day 7), and their corresponding three two-way interactions, and one three-way interaction. Models for both experiments were obtained by means of restricted maximum likelihood (REML) estimation, because it renders unbiased variance covariance parameters. The mean square (MS) and degrees of freedom (df) of the error terms were estimated following the more conservative Kenward-Roger method^32^. Significance of fixed effects, and their interactions, were obtained using Type II sum of squares. Homoscedasticity and normality of residuals of the models were visually checked and did not show considerable deviations from the model assumptions. All analyses were carried out using R version 3.6.3^33^, employing the packages lme4^34^, lmerTest^35^, lmtest^36^, pbkrtest^37^, car^38^, MuMIn^39^ and phia^40^. The proportion of variance explained by the model was obtained using the r.squaredGLMM function of the MuMIn package^41^, while the estimations of the magnitude effect for different predictors were obtained using the SS (sums of squares) provided by the function anova.lmer of the lmerTest package (Partial-eta^2^ according to the ratio SSeffect/[SSeffect + SSerror]). A posteriori tests were carried out using the false discovery rate correction with the p.adjust function of the base R package, in order to reduce the probability of obtaining a false positive (i.e., statistical significant test) when testing for differences between levels of factors.

## Results

**Figure 1:**
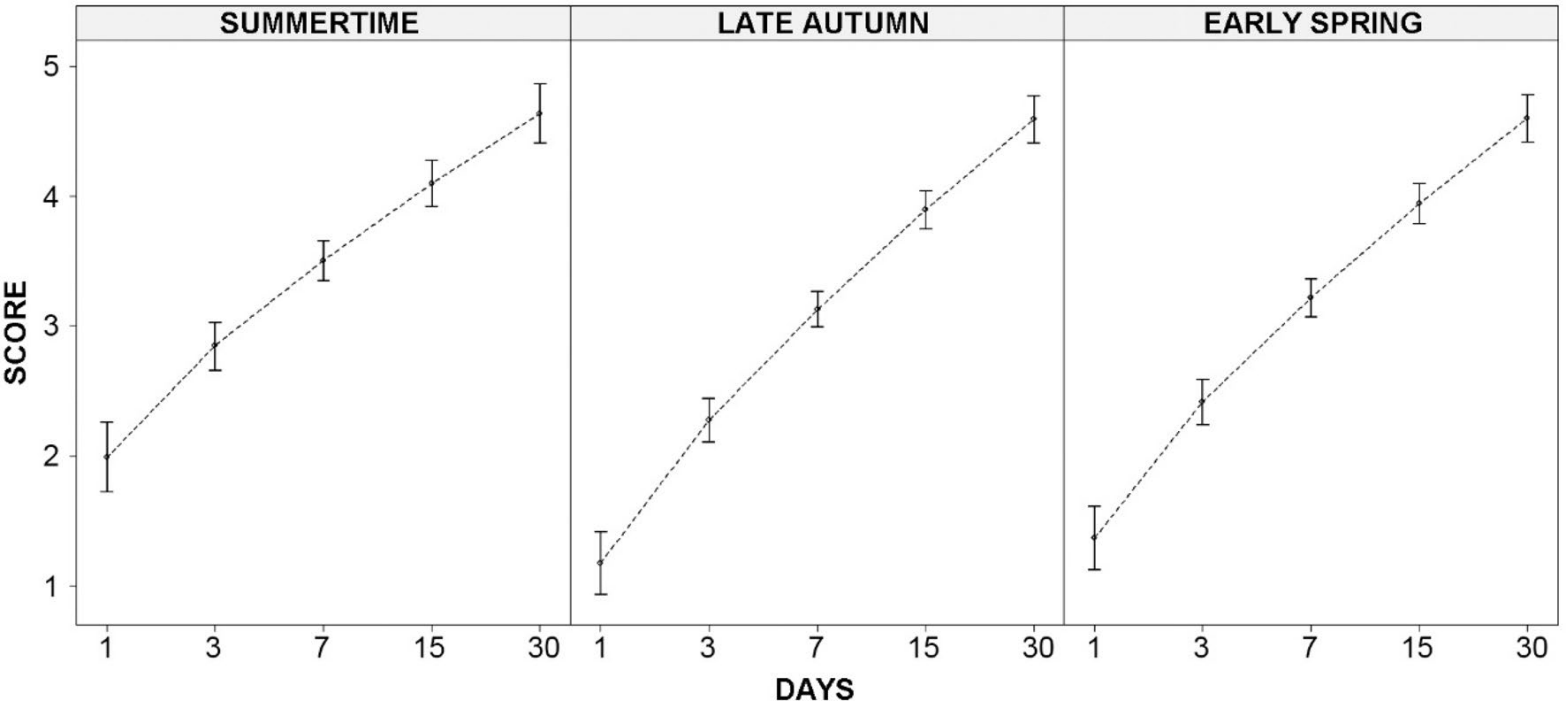
Decomposition scores of large-sized birds (i.e., hens) throughout time (five sampling events at days 1, 3, 7, 15 and 30 after the fresh carcasses were placed in the field) in three different seasons. The vertical bars show the average ± 95% confidence intervals of the data pooled for the two islands (the main effect of island, and the interactions between Island and the other two factors were not significant; see Table 2 for more detail).

Decomposition score of large birds (i.e., hens) across islands and throughout seasons and days after fresh carcasses were placed in the field was a highly explainable phenomenon (79.9% of the variance accounted for by the fixed effects, plus 8.9% attributable to random effects, in a highly significant three-way random intercepts and random slopes mixed model: χ^2^ = 380.6, df = 31, *P* < 0.001 derived from parametric bootstrap). Decomposition score significantly and monotonically increased with the logarithm of time since carcass placement (*P* ≪ 0.001; see Table 2 and Fig. 1). It was significantly different throughout seasons (*P* = 0.006), being larger in summer than in late autumn or early spring (*P *< 0.012 in the two a posteriori tests, with *P* = 0.751 for the autumn vs spring difference, using the false discovery rate significance correction). No statistical differences were found when comparing the two study islands. Only one out of the four interaction terms reached the significance level: days since placement by season (*P* = 0.006). The score was greater three and seven days after carcasses were placed in the field in summer than in the other two seasons (*P* < 0.030 in the six a posteriori tests using the false discovery rate for significance correction). The highest magnitude effect of the fixed terms included in the model, measured by the partial eta^2^, was obtained for the time after fresh carcasses were placed in the field (0.708), followed by considerably lower magnitude effects attributable to season (0.085) and the interaction time-season (0.053). Large birds that remained in the field for 15 days or more showed an average decomposition score ≥ 4, as post-colicuative or skeletal reduction (specially for 30 days), while those found with a score < 3 (nearly fresh or emphysematous) remained for less than 3 days (Fig. 1). Between 3 and 15 days after they were placed in the field (i.e., death), large birds showed a colicuative state (score = 3). Therefore, the decomposition score of carcasses provides an accurate clue to the time elapsed since the death of large birds. Scores larger than 4 are directly associated with deaths occurred at least 15 days before. In spite of a large temporal and environmental variation related to the experimental design, a very large proportion of the measurable variation in carcass state is related only to time since fatality. The carcasses decomposed more intensely during the first 3 days of stay in the field in the driest and hottest season of the year. Environmental differences attributable to the study region (two different islands) and sampling locations (random effects) were responsible of a very low proportion of variation in the decomposition state of carcasses.

**Table 2:** Three-way random intercept and random slopes mixed model analysing the decomposition score of large birds in three different seasons (summers, late autumn and early spring) and two study areas (islands of Lanzarote and Fuerteventura).

Fixed effects	p.eta^2^	SS	nDF	dDF	F	*P*
Days since placement (T)	91.6	0.708	1	13	510.4	≪ 0.001
Island (I)	0.1	0.002	1	14	0.8	0.396
Season (S)	3.6	0.085	2	10	9.1	0.006
Interaction T*I	0.3	0.007	1	14	1.8	0.199
Interaction T*S	2.0	0.053	2	101	5.4	0.006
Interaction I*S	1.3	0.334	2	10	3.4	0.073
Interaction T*I*S	0.6	0.015	2	101	1.5	0.221

p.eta^2^, Partial eta squared measuring the proportion of variance accounted for by a given fixed effect after accounting for that explained by other predictors in the model; SS, Sums of squares. nDF, Numerator degrees of freedom of the fixed effects (days since the placement of carcasses in the field has been treated as an ordinal factor, thus having one degree of freedom in the linear contrast of its levels). dDF, Denominator degrees of freedom using the Kenward–Roger method.

**Figure 2:**
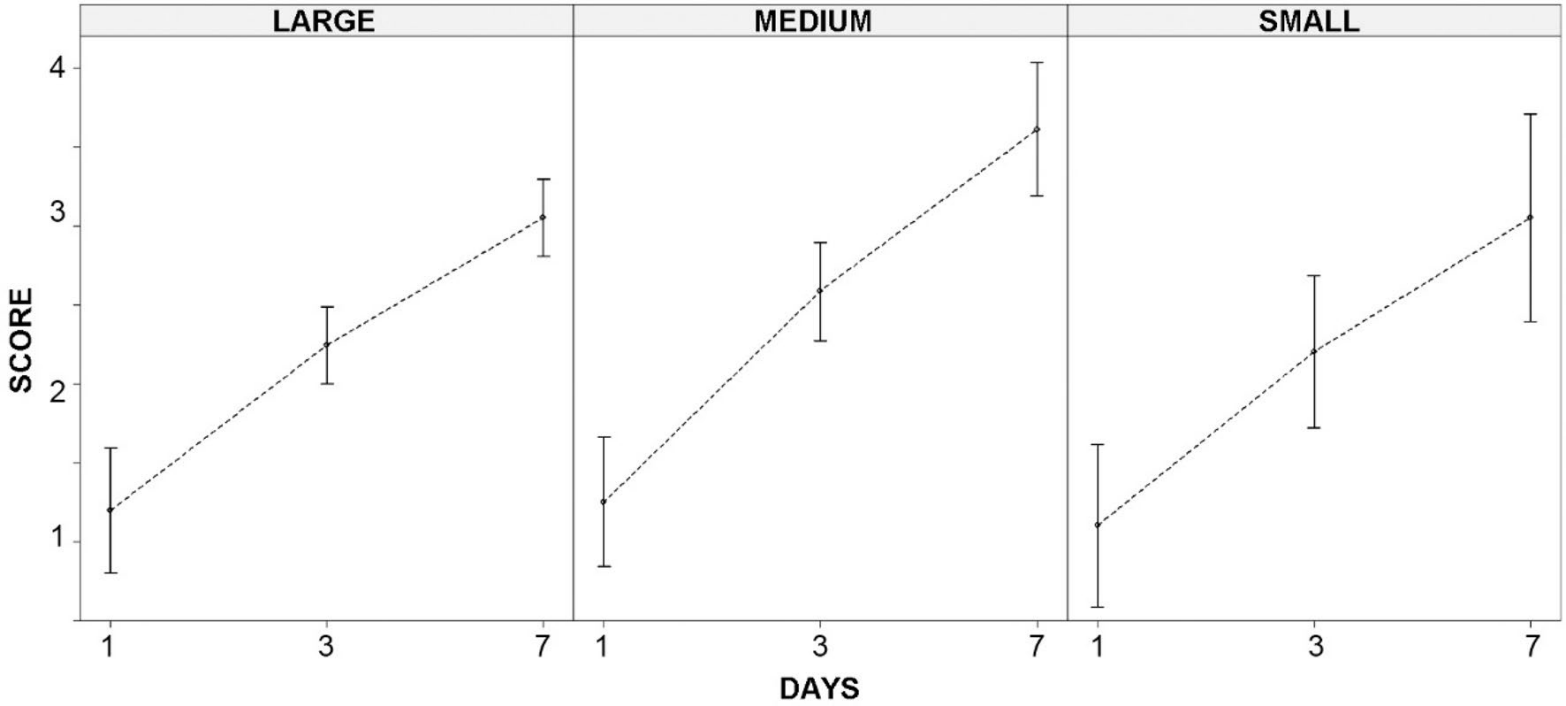
Decomposition scores of three different bird-sizes (large—domestic hens, medium—pigeons, small—chicks of domestic hens) throughout the first week after fresh carcasses placement in the field (three sampling events at days 1, 3, and 7) on the islands of Lanzarote and Fuerteventura. The vertical bars show the average ± 95% confidence intervals of the data pooled for the two islands (the main effect of island, and the interactions between Island and the other two factors were not significant; see Table 3 for more detail).

The analysis of the decomposition score of three body-size classes during the first 7 days after fresh carcasses were placed in the field in early spring shed light on differences in the decomposition rates related to bird body mass, and its interaction with the environmental variation associated with the two study islands and sampling locations within them. The three-way random intercepts and slopes mixed model was highly significant (χ^2^ = 122.8, df = 22, *P* < 0.001 derived from parametric bootstrap) and accounted for a relatively high proportion of variance: 60.6% attributable to fixed effects of island, carcass size, days since carcass placement in the field and their interactions, and 24.9% related to random differences among sampling locations. Only time elapsed since the fresh carcasses were placed in the field attained the significance level (*P* ≪ 0.001; Table 3 and Fig. 2), increasing linearly with the logarithm of time. Moreover, it attained the highest magnitude effect (partial eta^2^ = 0.476), in comparison with the other main effects and interaction terms (partial eta^2^ < 0.063). None of the comparisons between sizes within each sampling day in Fig. 2 reached the significance level (*P* > 0.268 in the nine a posteriori tests using the false discovery rate significance correction). Therefore, the increase in the decomposition score of carcasses with time during the first week was very similar irrespective of the size of the birds, not being higher than the colicuative state (score 3), and with a small proportion of variance accounted for by spatial location of carcasses (regarding the study region or the random effect linked to the position of the sampling areas).

**Table 3:** Three-way random intercept and slopes mixed model analysis of the decomposition score of three body-size classes during the first 7 days after fresh carcasses were placed in the field in early spring on the islands of Lanzarote and Fuerteventura.

Fixed effects	p.eta^2^	SS	nDF	dDF	F	*P*
Days since placement (T)	20.8	0.476	1	11	106.7	≪ 0.001
Island (I)	0.7	0.013	1	13	3.4	0.090
Size (S)	1.0	0.054	2	7	2.3	0.166
Interaction T*I	0.0	0.006	1	12	0.2	0.658
Interaction T*S	0.6	0.028	2	36	1.6	0.210
Interaction I*S	1.0	0.063	2	8	2.3	0.158
Interaction T*I*S	0.3	0.020	2	36	0.8	0.455

For more details see Table 2.

## Discussion

This work provides a useful methodology for the estimation of the time since fatalities with manmade structures that could be used during the estimation of bird collision rates. By means of two decomposition rate experiments, we gained insights into the spatial, temporal and seasonal variation of the decomposition process of carcasses. Our results suggest that the score of decomposition is positively and monotonically affected by the time since carcass placement in the field, and it is larger during dry and hot seasons (i.e., summer) as compared with wet and mild or cold seasons (i.e., late autumn and early spring). Indeed, the decomposition score increased more slowly over time in the driest and hottest season (summer), because the degradation score during the first week was larger. These patterns are very consistent across spatial scales or animal body sizes, as the decomposition score did not vary between islands and among locations within islands, or among carcass sizes up to 7 days since carcass placement (i.e., fatalities). Future studies aiming to estimate bird collision rates with power lines and other infrastructures may found it useful to apply the experiments carried out in this work in order to elucidate the decay rate of carcasses as well as the factors influencing it.

Decomposition stages occur sequentially as the time since death evolves, from algor, livor and rigor mortis, to desiccation, mummification and skeletization^12^. Therefore, it is reasonable that the decomposition scores increased with the time since carcass placement, being the factor with the highest magnitude effect in this study. A carcass with a decomposition score ≥ 4 (post-colicuative or skeletal reduction) had been in field for 15 days or more, whereas a carcass with a score < 3 (fresh or emphysematous) had been exposed for less than 3 days (Figs. 1, 2). These results are consistent with the only two bird decomposition experiments carried out previously, using Common kestrel (*Falco tinnunculus*)^17^ and domestic chicken carcasses^21^. In these studies, gas production (emphysematous stage) started 1–3 days after carcass placement, whereas the skeletonization stage was reached at 11–15 days^17,21^.

Although decomposition score is mainly associated with carcass age, there are other sources of variation that also influence the decomposition process, namely environmental conditions related to temperature, solar radiation, precipitation, or moisture^12,24,42^. In our study, these individual effects were related to season: the decomposition scores were higher in summer than in late autumn or early spring. Temperature highly determines the speed of the decay process as well as the duration of each decomposition stage^28,43^. Indeed, the accelerated effect of temperature on decay rates^42^ might partially explain the higher decomposition scores observed in summer, as high temperatures favour insect activity, bacterial growth and the development of dipteran and coleopteran larvae, facilitating putrefaction^16,24,44^. Moreover, photodegradation can breakdown resistant substrates and promote microbial activity up to a threshold from which solar radiation may negatively impact on microbial growth and activity at daytime^45–48^, although microbial degradation may continue at night-time (see Gliksman et al.^29^ and Araujo et al.^30^ for litter decomposition in drylands). On the other hand, humidity has been described to increase insect activity and delay desiccation^28^, thus favouring the consumption of tissues and hastening carcass decomposition^25^. Finally, rainfall, wind and other unfavourable weather conditions may prevent or reduce insect activity, thus slowing down decomposition rates^28^. Overall, season acts as a surrogate of temperature, sun exposure, moisture and precipitation, conditioning the different decomposition scores observed throughout the year in this study. Regarding the spatial pattern of the decomposition process, the small proportion of the variance explained by islands (fixed factor) or sampling sites (random effect) suggests that there is little spatial variability across the study area in the influence of these abiotic factors, despite the large area over which the experiment was carried out (125 km between the most distant sampling locations over an area of ca. 2500 km^2^). However, it should be noted that our study area has a reduced variety of habitats (lava fields, dunes, semi-arid and arid habitats), within a small altitudinal gradient that generates little climatic variation. If the other study areas subjected to sampling occupy huge extensions of land, with a great variety of habitats and a wide elevational gradient, the differences between localities in the decomposition-degradation states could be greater as a consequence of a considerable variation in temperature and humidity. On the other hand, the decomposition process would have been slower in colder regions than our study area, with lower decomposition-degradation scores for the same dates after death (e.g.,^14,19^). In this case, it would be advisable to carry out several pilot studies that characterize the decomposition rates under that great diversity of environmental conditions.

In disagreement with expectations, decomposition rates during the first week after placement did not differ between carcass sizes related to body mass of species. Previous studies have observed inconsistent results on the effect of body size on the rate of decomposition. Faster decomposition rates have been reported in carcasses of obese humans as compared to smaller ones (e.g., skinny bodies or new-borns), probably due to the higher content of liquids from the liquefaction of body fats^28,44^. Similarly, Cambrá-Moo et al.^21^ found that older and bigger individuals of domestic chicken decayed faster in the early stages of decomposition. Conversely, other studies have reported that decomposition rates are slowed down in big carcasses due to the higher content of fat and flesh, and lower heat loss^49–52^. In particular, Sutherland et al.^16^ reported similar decomposition rates during the first stages, but with faster decomposition rates for small-sized carcasses during later stages; consistently with this work, we observed similar decomposition rates up to 7 days after carcass placement. The high rate of carcass disappearance of small and medium-sized carcasses due to scavenger removal^8^ leads to analytical power limitations derived from small sample sizes, which made it impossible to assess the evolution of the decomposition scores up to 30 days after placement. However, differences on decomposition rates among body sizes might be detectable at higher decomposition scores (≥ 4 or *post-colicuative*) when all soft tissues have been consumed by insects or microorganisms, or they have become desiccated, because soft tissues can be consumed before they become desiccated in small-sized carcasses. Some carcasses are not completely removed by scavengers and may still be detected in the field as “partial carcasses”^53^. There were no partial carcasses in our study, but we think that even with those partial remains it would still be possible to estimate the time since death, according to the physical descriptions in Table 1. Future decomposition experiments should protect carcasses from predation (e.g. Valverde et al.^17^) in order to evaluate this effect in the long-term.

The estimation of the post-mortem interval is crucial to accurately assess bird collision rates with manmade structures if the carcasses cannot be initially removed from sampling areas due to logistical difficulties or legal issues before carcass search begins. In this study, we provide a cost-efficient protocol, compatible with the classical methodologies based on the prior removal of carcasses, which allows the decomposition score to be linked as unambiguously as possible to the most likely elapsed time since the fatality occurred (e.g. collision with a power line). We suggest that experiments like those carried out in this paper should be conducted prior to the survey of manmade structures in search of bird fatalities, with the aim of understanding how the decomposition process proceeds in a given study area and under specific environmental conditions. Afterwards, only those carcasses that can be assigned to a reliable time interval should be considered in the estimation of the mortality rates.

## Supplementary Information

Supplementary Information 1.

Supplementary Information 2.

## Data Availability

Data used in the current study is available as supplementary material.
