# Supplementary material for: Decomposition stages as a clue for estimating the post-mortem interval in carcasses and providing accurate bird collision rates
Source: Sci Rep. 2022 Sep 28;12:16188. doi: 10.1038/s41598-022-20628-3 (PMC9519910; doi:10.1038/s41598-022-20628-3)
Supplement: Supplementary file 2 — Supplementary Information 2. [file 41598_2022_20628_MOESM2_ESM.docx]

**SUPPLEMENTARY ELECTRONIC MATERIAL**

**Decomposition stages as a clue for estimating the post-mortem interval in carcasses and providing accurate bird collision rates**

Virginia Moraleda^1^, Julia Gómez-Catasús^2,3,4^, Claudia Schuster^1^, Luis M. Carrascal^5^

^1^ Grupo de Rehabilitación de la Fauna Autóctona y su Hábitat, GREFA, Majadahonda, Madrid, Spain.

^2^ Novia University of Applied Sciences, Raseborgvägen 9, FI-10600, Ekenäs, Finland

^3^ Terrestrial Ecology Group, Department of Ecology, Universidad Autónoma de Madrid (TEG-UAM), 28049 Madrid, Spain.

^4^ Centro de Investigación en Biodiversidad y Cambio Global, Universidad Autónoma de Madrid (CIBC-UAM), 28049 Madrid, Spain.

^5^ Departamento de Ecología Evolutiva, Museo Nacional de Ciencias Naturales (MNCN-CSIC), 28006 Madrid, Spain.

**APPENDIX A. Description of the decomposition states of carcasses**

In this Appendix we describe the decomposition scores or states provided in this study. We incorporate information regarding the physical situation of the carcass but also about the cadaveric fauna found. However, is it important to highlight that in order to make a specific dating, a detailed entomological study is needed, in addition to establishing specific climatic conditions and knowing the invertebrate species present in the study area in the different seasons. See Barnes^1^, Fernández^2^ and Valverde *et al.*^3^ for similar descriptions on the cadaveric fauna found.

**State 1 or fresh** was characterized by the presence of soft tissues, covering from death to body inflammation due to bacterial fermentation (Fig. 1). The first signs of decomposition appear in the abdominal area where, due to hemoglobin degradation, a greenish coloration is seen in the skin and adjacent tissues. Indications of the freshness of the corpse were, for example, the presence of fresh blood or the good condition of feathers that do not easily detach from the body. Flies and wasps were the initial colonizers of the carcasses, but also beetles, ants and eggs deposited in areas such as oral cavity, eyes, open wounds, or cloaca. In some cases, we also found first instar larvae.

**
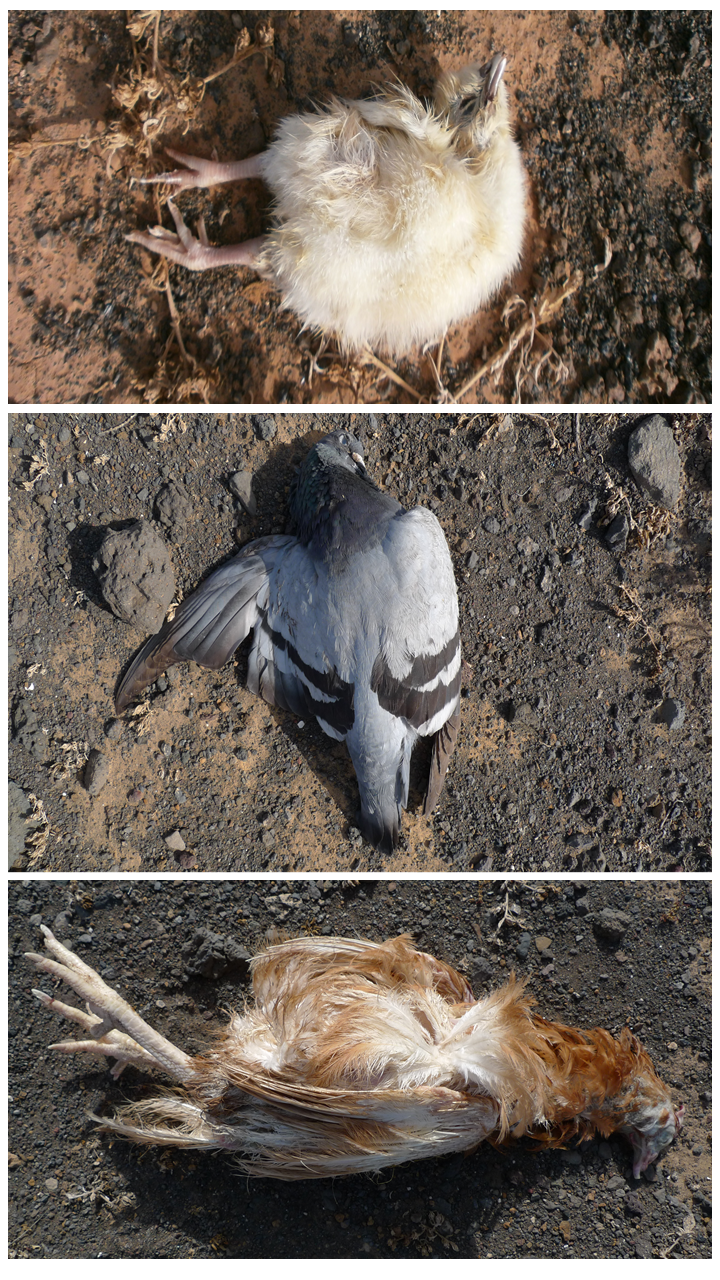
**

**Figure A1.** From top to bottom: carcass of chick of Domestic Chicken *Gallus gallus domesticus*, Rock Pigeon *Columba livia* and Domestic Chicken in decomposition state 1 or fresh

**State 2 or *emphysematous*** included from the beginning of inflammation caused by bacterial fermentation until body rupture due to pressure and superficial tissue decomposition (Fig. 2). This period is characterized by the presence of gas from bacterial decomposition, which accumulates in the digestive tract and other body cavities, resulting in great distension of the corpse. Thus, the corpses were found swollen, and in some cases fresh blood was still visible. Regarding the cadaveric fauna, we still found ants, beetles and flies in adult stages, but also in egg stages and increase the proportion of larvae which were found at different stages and therefore of different sizes.


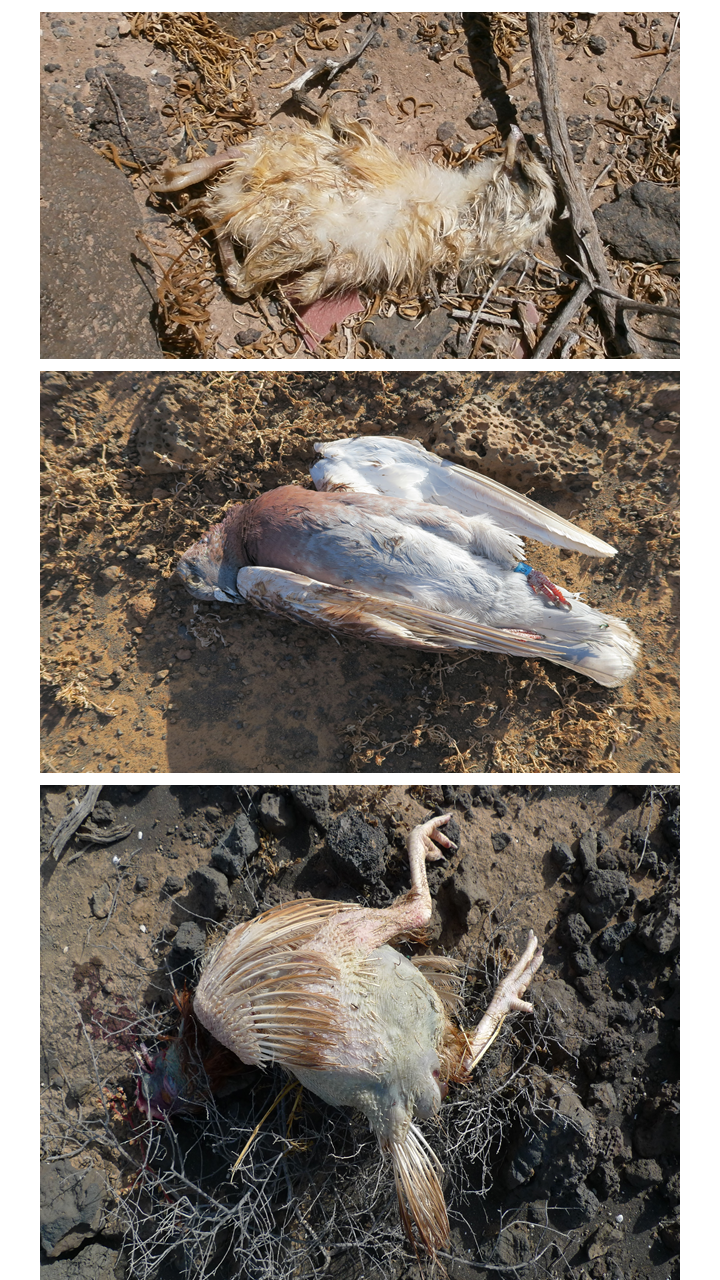


**Figure A2.** From top to bottom: carcass of chick of Domestic Chicken, Rock Pigeon, and Domestic Chicken in decomposition state 2 or *emphysematous*.

**State 3 or *colicuative*** encompassed from gas outlet until decomposition and disappearance of soft tissues (Fig. 3). In this phase the epidermis detaches from the dermis and liquefaction of the soft tissues and softening of all organs occurs. At this stage little skin remains, and the bones and tendons are still moist. The feathers are in bad conditions or totally degraded in some parts. We found a higher proportion of beetles than in previous states, and to a much lesser extent flies and ants. This is the most active phase of the larvae of necrophagous insects and thus, many larvae were found in different stages (and sizes) and pupating individuals appear for the first time.


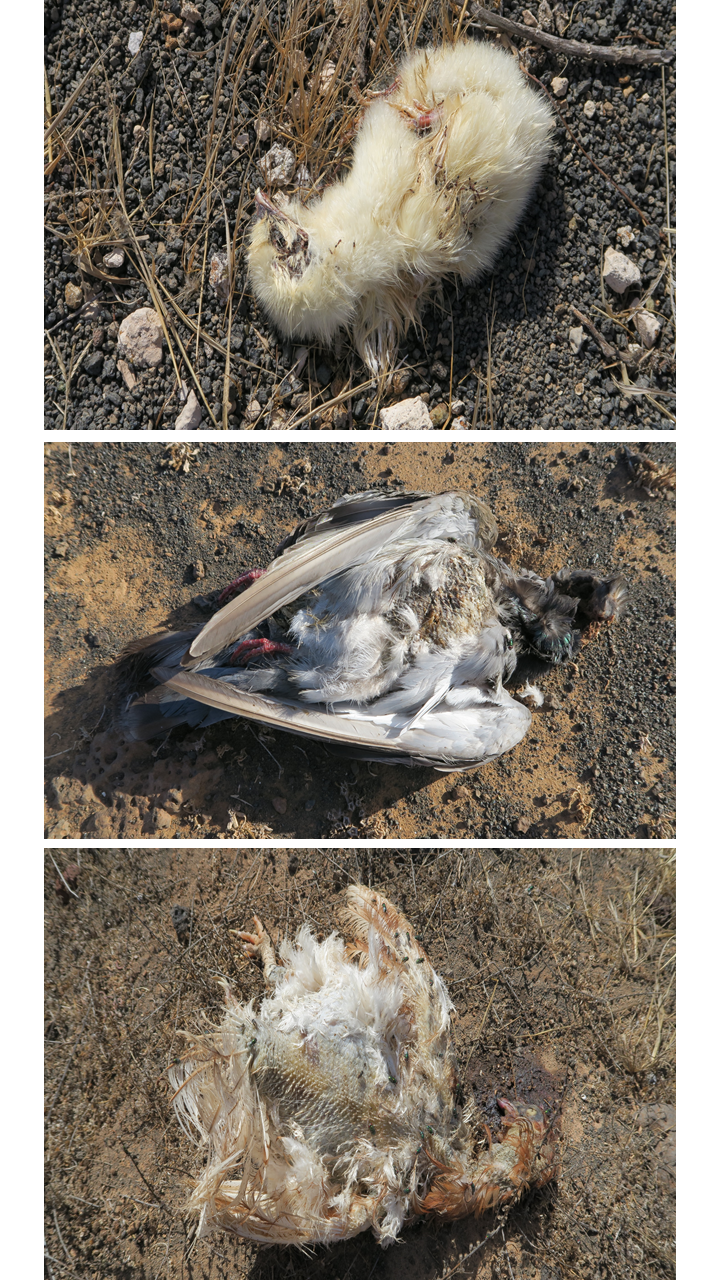


**Figure A3.** From top to bottom: carcass of chick of Domestic Chicken, Rock Pigeon, and Domestic Chicken in decomposition state 3 or *colicuative*.

**State 4 or *post-colicuative*** the bodies were hollow and mummified. Only dried tissues, cartilages and bones were present (Fig. 4). In this stage we observed mainly adults of beetles, but not ants. We also found individuals in pupal stages and, to a lesser extent, in larvae stages. In some occasions, we encountered individuals of Diptera starting a new life cycle (adults, eggs and larvae).


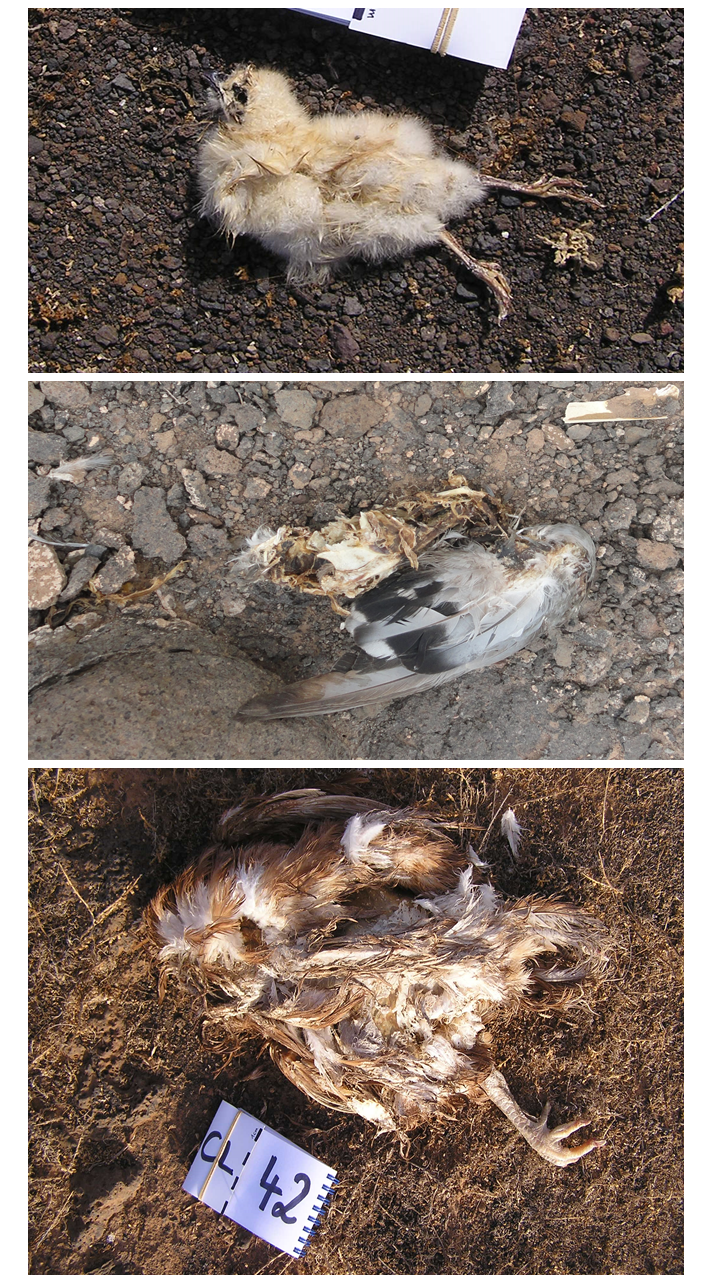


**Figure A4.** From top to bottom: carcass of chick of Domestic Chicken, Rock Pigeon, and Domestic Chicken in decomposition state 4 or *post-colicuative*

**State 5 or *skeletal reduction*** occurred when only bone remains were distinguishable (Fig. 5). The cadaveric fauna significantly decreased. We observed mainly beetles, flies disappeared completely and in some rare cases we saw individuals in larval or pupal stages. At this stage, mites and arachnids appeared.


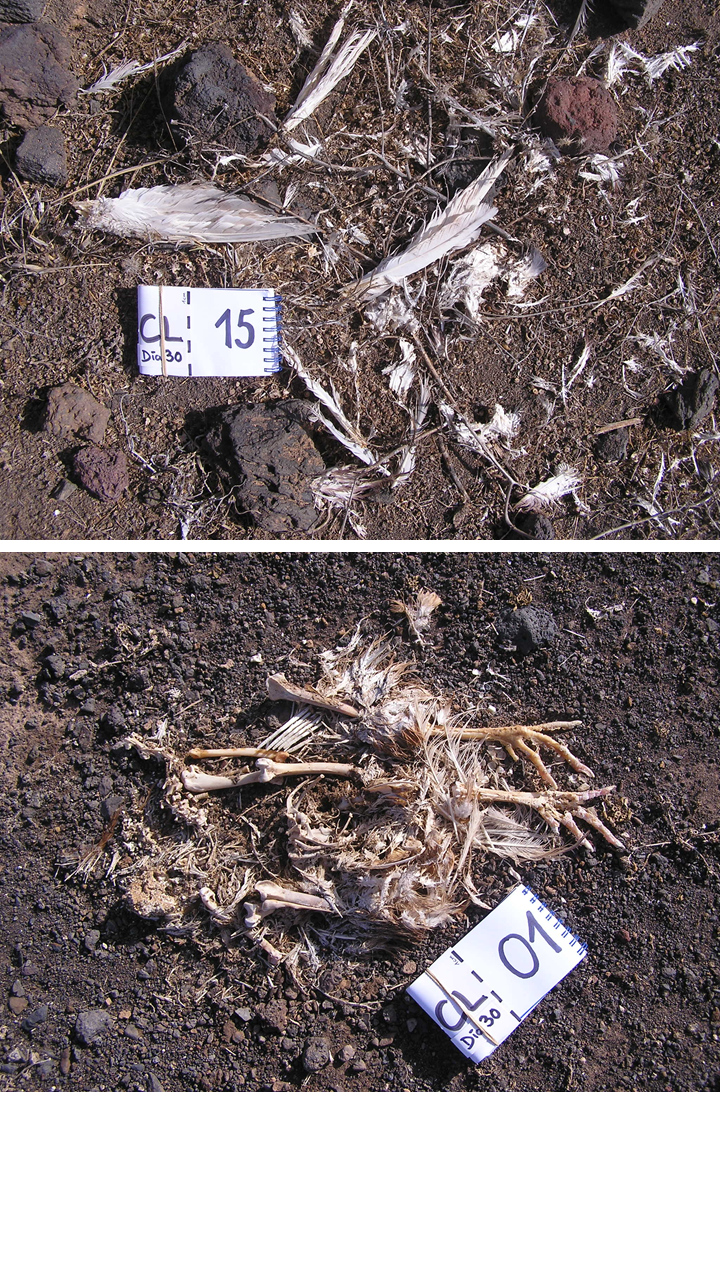


**Figure A5.** From top to bottom: carcass of Rock Pigeon, and Domestic Chicken in decomposition state 5 or *skeletal reduction*. We did not find carcass of chick of Domestic Chicken in decomposition state 5.

**References**

1. Barnes, K. M. in *Wildlife forensic investigation: principles and practice* (eds. Cooper, J. & Cooper, M.) 149–160 (CRC Press, 2013).

2. Fernández, I. *Datación de la muerte del cadáver y entomología cadavérica. In: Manual de protección legal de la biodiversidad para los agentes de la autoridad ambiental en Andalucía*. (Consejería de Medio Ambiente y Ordenación del Territorio, Junta de Andalucía, 2015).

3. Valverde, I., Espín, S., María-Mojica, P. & García-Fernández, A. J. Protocol to classify the stages of carcass decomposition and estimate the time of death in small-size raptors. *Eur. J. Wildl. Res.* **66,** 1–13 (2020).
